# Somatosensory cortex shapes perceptual decision bias via the superior colliculus

**DOI:** 10.21203/rs.3.rs-7742853/v1

**Published:** 2026-06-22

**Authors:** Alice Y. Nam, Jiwook Shin, Morgan Tenney, Baihe Zhang, Y. Kate Hong

**Affiliations:** 1 Department of Biological Sciences, Carnegie Mellon University, Pittsburgh, PA 15213, USA; 2 Neuroscience Institute, Carnegie Mellon University, Pittsburgh, PA 15213, USA

## Abstract

Sensory inputs reach cortical and subcortical areas in parallel, but how they coordinate to guide decisions is not well understood. Here, we examined primary somatosensory cortex (S1) and superior colliculus (SC) in mice performing a tactile detection task. Inactivating S1 contralateral to the stimulus decreased the detection probability as expected. Surprisingly, inactivating ipsilateral S1 increased detection probability, even when the stimulus was absent. These opposite effects reflected shifts in choice bias, rather than changes in sensitivity. SC inactivation produced similar directional bias shifts, and SC lesions abolished the effects of S1 inactivation. Neural recordings showed that although S1 inactivation decreased SC sensory responses, choice-activity remained robust in SC, but fell to chance in S1, suggesting that behavioral impairment was better explained by changes in SC-dependent choice signals rather than the loss of S1 sensory encoding. Thus, S1 shapes tactile behavior by modulating SC-dependent circuits that help transform sensory evidence into a decision.

## INTRODUCTION

Perceptual decisions depend not only on the fidelity of sensory encoding, but also on how sensory evidence is weighed against internal decision criteria. Causal perturbations are prevalently used to infer the functional necessity of a brain region. However, behavioral deficits that accompany such perturbations can be difficult to interpret. For example, inactivation of a cortical area may impair behavioral performance by reducing perceptual sensitivity, but could also alter choice bias, defined as the systematic tendency to favor one behavioral response over another independent of sensory evidence. Distinguishing between the possibilities is critical for understanding how sensory areas contribute to perception.

The primary somatosensory cortex (S1) is the main cortical target for tactile input. Across primates and rodents, S1 perturbations severely impair discrimination of fine tactile features such as texture, shape, and object location^1–8^, whereas the effects on simple tactile detection is less clear^6,8,9^. In the rodent whisker system, acute S1 inactivation only partially impairs tactile detection^9–12^, and chronic whisker S1 lesions lead to rapid recovery^3,6,8,9,13,14^, suggesting that alternative pathways can support detection behavior^15^.

The superior colliculus (SC) is well-positioned to mediate tactile detection behavior. Although mostly studied for its roles in vision, eye movements, and head-orienting, the SC is an evolutionarily conserved midbrain structure that mediates rapid detection of multimodal stimuli to coordinate behavioral responses. Beyond its role in sensorimotor transformation, recent work highlights SC’s expanded role in more cognitive processes, including attention and perceptual decision-making^16–23^. In addition, relative differences in activity between two SC hemispheres have been proposed to account for perceptual decisions during a visual attention task, suggesting that interhemispheric SC balance may contribute to choice bias^24^.

SC neurons in the intermediate and deep layers respond to somatosensory stimuli^25–27^ and provide a subcortical pathway that could mediate tactile detection in the absence of S1^15^. Primary sensory cortices, including visual and whisker S1, directly and indirectly project to SC, where they have been shown to modulate sensory gain or drive SC-mediated behavior^28–31,31,32^. However, how primary sensory cortices and SC coordinate activity to guide behavior remains poorly understood.

Here, we examined how S1-SC interactions contribute to shaping perceptual choices. In mice performing a whisker-mediated tactile detection task, contralateral S1 inactivation impaired behavioral performance, not simply by decreasing sensitivity, but by shifting choice bias. Neural recordings revealed that S1 inactivation reduced tactile stimulus encoding in SC and altered the balance of activity across SC hemispheres. Moreover, the stimulus-evoked SC activity remained strongly predictive of trial-by-trial choice, even when S1 was silenced, and disrupting SC abolished the behavioral effect of S1 inactivation. Together, these results are consistent with a model in which S1 contributes to tactile detection not only by encoding sensory information, but by modulating SC-dependent circuits that determine how tactile evidence is converted into perceptual decisions.

## RESULTS

### Contralateral S1 inactivation shifts decision bias but not sensitivity

**Extended Data Fig 1. |:**
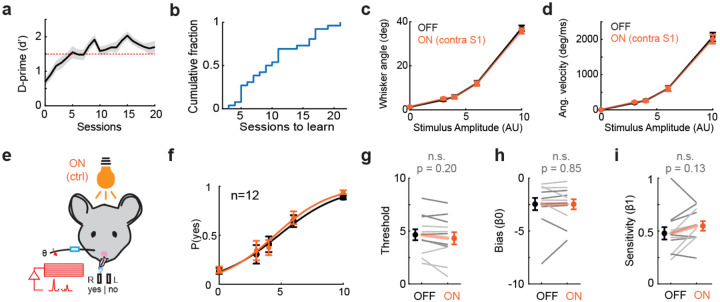
Learning, whisker stimulus calibration, and negative controls. **a,** Performance measured by d-prime (d’, amp 0 vs. amp 10) over learning sessions for animals used in this study (n=26 mice). Learning criterion was defined as d’ greater than 1.5 (red dotted line) over 3 consecutive sessions. **b,** Cumulative plot of data in a. **c,** Whisker deflection angles for each stimulus amplitude for S1 inactivation experiments in Fig.1. (n=22 sessions, 6 mice), for laser OFF (black) vs. ON (orange) trials. **d,** Whisker deflection measure in angular velocity. **e-i,** Laser only negative controls for S1 inactivation experiments; 593-nm laser was visible but did not alter S1 (or SC) activity. **f,** Probability of detection, P(yes), by stimulus amplitude. Solid lines are logistic fits of average responses at each amplitude for OFF vs. ON trials; n=12 mice. **g** Detection threshold. **h,** Bias; **i,** Slope. n=12 mice (6 mice from Fig. 1, lighter gray; 6mice Fig.3, darker gray). Error bars: median ± interquartile range; **g-j**, Wilcoxon signed rank test; n.s., not significant.

**Fig. 1 |:**
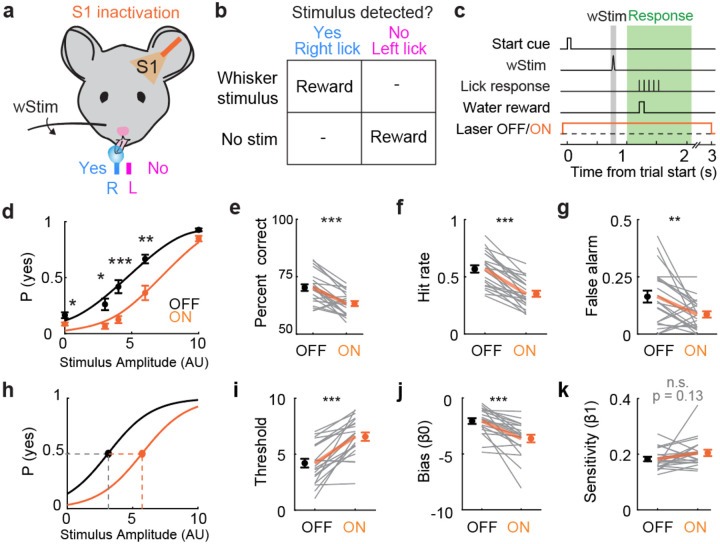
S1 inactivation impairs detection due to a shift in decision bias. **a,** Head-fixed mice were trained to report the detection of a tactile stimulus delivered to a single (right, C2) whisker (wStim). **b,** Mice respond by licking right to report stimulus detection (‘yes’) or left to report (‘no’) and received a water reward for correct responses. **c,** Trial structure: an auditory start cue was followed by a whisker stimulus of amplitude 0–10 arbitrary units (AU, 0–40° downward deflection, ~50ms pulse). After a 300ms delay, the response window opened (2s), followed by a 3s inter-trial interval. Contralateral S1 was optogenetically inactivated (ON) on randomly interleaved trials for the full trial duration. **d,** Probability of ‘yes’ response (P(yes)) across stimulus amplitudes (OFF, black; ON, orange); **e,** Percent correct; **f,** Hit rate; **g,** False alarm rate; **h,** Example psychometric curves from one session with detection threshold indicated by dashed lines; **i,** Detection threshold (*p = 1.1e-4*). **j,** Bias (β_0_) shifted with inactivation (*p = 6.4e-37*); **k,** Sensitivity (β1, *p= 0.11*). Error bars: mean ± s.e.m.; * *p*<*0.05*, ** *p*<*0.01*, *** *p*<*0.001, n.s., p*>=*0.05*. **d-i**, two-sided Wilcoxon signed-rank test; multiple comparisons across amplitudes corrected by Bonferroni; **j-k**, trial-level generalized linear mixed-effects models (LME; fixed effect: laser; random effects: session nested in animal); n=22 sessions; 6 mice.

To determine the role of S1 in perceptual decision-making, we trained animals on a head-fixed, whisker-mediated tactile detection task. To minimize confounds from variability in whisking, we used passive stimulation of the ‘C2’ whisker on the animal’s right side with a single downward deflection, sampling a broad range of stimulus amplitudes to span high- and low-salience stimuli to ensure comprehensive coverage of the perceptual range (0–10 arbitrary units (AU), calibrated to a maximum 40° whisker angle, or ~2100 °/s) (Extended Data Fig. 1c, d). We implemented a forced-choice (Yes/No) paradigm, in which animals indicated their choice by licking one of two lick ports: right to respond ‘Yes’ or left to respond ‘No’ to stimulus detection, and receiving water rewards for correct responses (Fig. 1a, b). This forced-choice paradigm requires an overt response on every trial, allowing changes in detection behavior to be distinguished from failures to respond.

Tactile inputs from one side of the body cross the midline at the level of the brainstem to reach the contralateral thalamus and S1^33,34^. Here, we refer to *contralateral* and *ipsilateral* sides relative to the tactile stimulus, which was always delivered to the right side of the animal. We optogenetically silenced contralateral S1 (S1_contra_) in *Emx1-Halo* mice, which express inhibitory halorhodopsin (*eNpHR3.0*) in ~80% of excitatory cortical neurons^9,35^. Consistent with numerous studies^9,4,5,11,10,6,3,14^, acute S1_contra_ suppression (laser ON) decreased overall % correct performance (Fig. 1e) driven by an overall decrease in ‘Yes’ responses across stimulus amplitudes (Fig. 1d), including no-stimulus trials (Fig. 1g, false alarms, *p = 0.005*, two-sided Wilcoxon signed-rank test). Because the forced-choice task design requires animals to always report their decision, the global decrease in ‘Yes’ responses was not simply due to disengagement or impaired ability to respond during laser ON trials. In laser control experiments, in which the laser illumination was visible but did not alter S1 activity, no changes in behavior were observed (Extended Data Fig. 1c–d).

**Extended Data Fig. 2 |:**
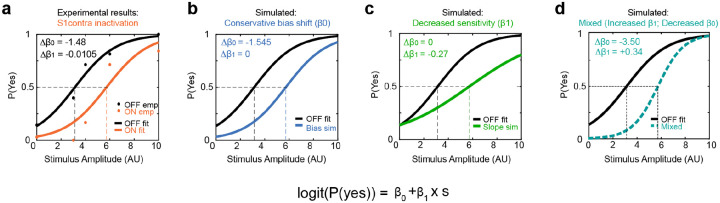
Illustration of threshold changes due to sensitivity vs. bias. Psychometric functions were modeled using logistic regression to illustrate how bias (β_0_) and sensitivity (β_1_) parameters influence detection threshold. **a,** Example session from Fig. 1h showing empirical data (emp, filled dots) and logistic regression fits for laser OFF (black) and ON (orange) trials. OFF-trial empirical example data was used as baseline for all panels A-D. Threshold (dashed lines) is equivalent to the stimulus amplitude needed for a 50% ‘yes’ response rate (P(Yes) = 0.5, given by -β_0_/β_1_). **b,** Simulation of only changing the bias (Δβ_0_) to produce the same shift in detection threshold, which shifts the curve laterally without altering slope (blue). **c,** Simulation of a sensitivity change (Δβ_1_) without bias change, which decreases the slope to reach the same threshold (green). **d,** simulation showing how increases in slope (combined with bias shift) can also reflect impaired performance (dashed teal line). These examples illustrate that either bias or sensitivity can affect threshold, but the empirical example is most correlated with a shift in bias.

To further dissect the observed changes in threshold, we generated psychometric curves by fitting logistic regression models to trial-by-trial responses as a function of stimulus amplitude for each session (Fig. 1h). As expected^9,11,13^, S1_contra_ inactivation significantly increased the detection threshold, equivalent to the stimulus amplitude needed to respond ‘Yes’ 50% of the time (Fig. 1h–i). To further dissect how S1 inactivation altered psychometric performance, we considered two logistic regression parameters: decision bias (β_0_, intercept) and sensitivity (β_1_, slope), which together determine the detection threshold (illustrated in Extended Data Fig. 2, Equation (1)). *Decision bias* reflects the subject’s overall tendency to favor one choice over another (e.g., Yes vs. no), independent of stimulus strength, and is reflected by a lateral shift in the psychometric curve; *sensitivity* captures how reliably choices scale with changes in stimulus amplitude, and is reflected by the slope of the psychometric curve.

S1_contra_ inactivation induced a significant decrease in decision bias (Fig. 1j; *p = 7.4e-6*). This is consistent with a conservative bias shift, in which subjects are less likely to respond ‘Yes’, regardless of the stimulus amplitude. In contrast, average sensitivity did not differ, with S1 inactivation leading to small or inconsistent changes across sessions (Fig. 1k; *p = 0.13*, Wilcoxon signed-rank test). Since both increases and decreases in slope can reflect impaired performance (Extended Data Fig. 2), average sensitivity measurements were difficult to interpret. We therefore focused subsequent analyses on detection threshold and bias shifts, which offered more robust and interpretable measures of behavioral change. Together, these results indicate that the primary effect of S1_contra_ inactivation was to increase detection threshold due to systematic shift decision bias, rather than a reliable change in sensitivity.

### SC_contra_ inactivation shifts choice bias, similar to S1_contra_ inactivation

**Fig. 2 |:**
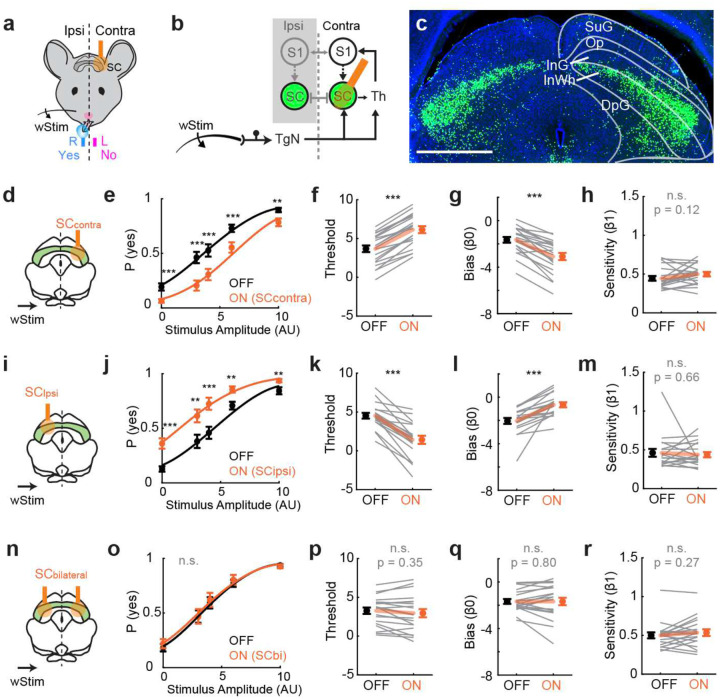
Inactivation of the superior colliculus (SC) directionally shifts action selection. **a,** Schematic of Yes-No detection task in *Pitx2-Halo* mice with SC inactivation**. b. Simplified** circuit diagram **highlighting major somatosensory pathways to SC.** Whisker stimuli (wStim) are transduced via the trigeminal ganglia and relayed through the trigeminal brainstem nuclei (TgN), which project to SC, and indirectly to S1 via the thalamus (Th). S1 projects both directly and indirectly to SC in the same hemisphere; SC, in turn, projects to the secondary somatosensory thalamus. **c,** *Pitx2-Halo* mice were used to silence excitatory neurons in the intermediate SC, where whisker-responsive and premotor neurons are located (image from *Pitx2-GFP* showing targeted cells). **d-h,** Effects of SC_contra_ inactivation (n= 21 sessions, 6 mice; **e,** logistic fits of average probability of ‘yes’ response (P(yes)), **f** detection threshold; **g**, decision bias (β_0_); **h**, sensitivity (β1). **i-m,** Same as d-h but for SC_ipsi_ inactivation (n= 19 sessions, 6 mice); **n-r,** for bilateral SC inactivation (n= 20 sessions, 6 mice). Abbreviations: superficial gray (SuG), optic layer (Op), intermediate gray (InG), intermediate white (InW), Deep SC: deep gray (DpG). Scale bar: 1mm. Error bars: mean ± s.e.m.; P(yes) and threshold: two-sided Wilcoxon signed-rank test (Bonferroni correction for e, j, o). Bias and sensitivity were assessed with LMEs.

The intermediate layers of the SC (Fig. 2b–c) receive direct ascending input from the contralateral trigeminal brainstem nuclei, providing a direct pathway for bottom-up tactile information, independent from S1^29,30^ (Fig. 2b). Previous work has shown that SC can partially mediate tactile behaviors in a conditioned foot-shock avoidance task^15,36^, consistent with SC’s general role in mediating stimulus-induced escape behaviors^16,36,37^. This raises the possibility that SC may also contribute directly to perceptual decisions during operant tasks.

**Extended Data Fig. 3 |:**
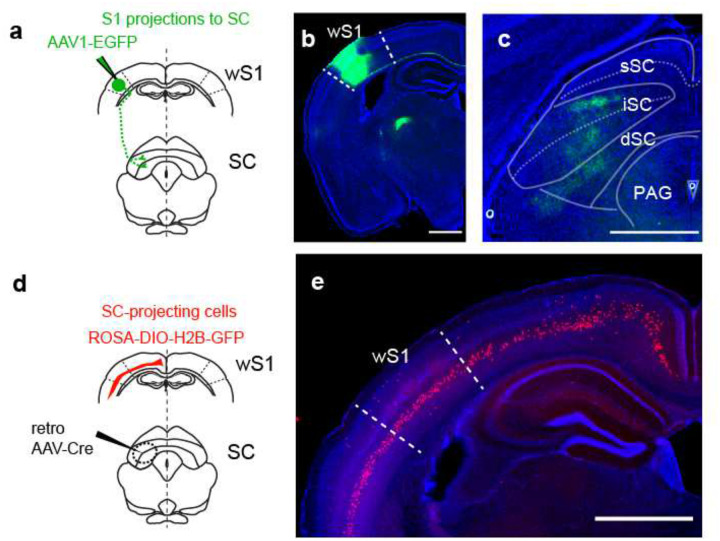
Whisker S1 projects to intermediate and deep SC. **a,** schematic of injection**, b,** AAV1-EGFP was injected into the C2 barrel in whisker S1 (wS1) to trace S1 axon projections to SC. **c,** S1 axon terminals are found in the central upper and lower patches of the intermediate SC layers (iSC), as well as deep SC (dSC). No axons were found in the opposite SC (not shown). **d**, Schematic of retrograde AAV-Cre injection into SC in an H2B-GFP reporter line, to label S1 neurons bodies that project to SC**. e,** Retrogradely labeled cortical neurons are found primarily in L5b in wS1 and broadly across cortex, in red. wS1: whisker S1 (barrel cortex) sSC: superficial SC; iSC: intermediate SC; dSC: deep SC layers. Scale bars: 1mm.

**Extended Data Fig. 4 |:**
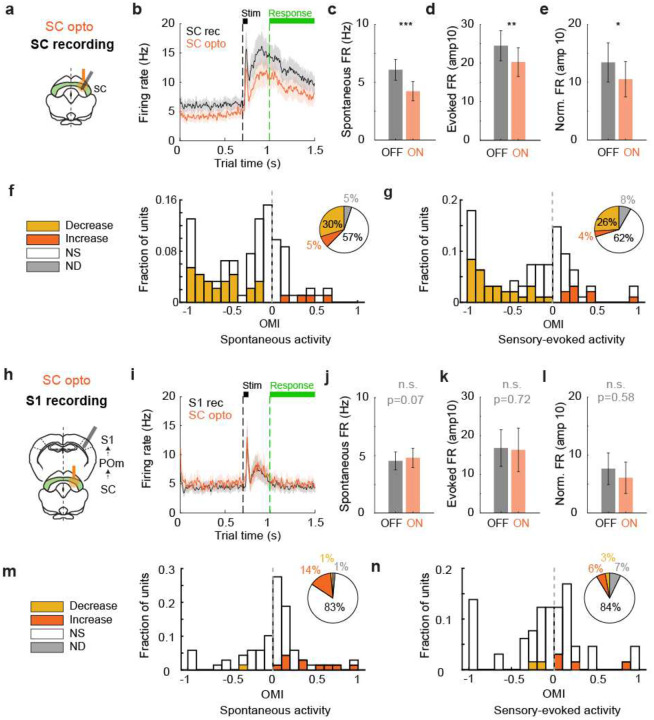
SC and S1 recordings during inactivation of intermediate SC in *Pitx2-Halo* mice. **a-g**, SC recording during SC inactivation (2 *Pitx2-Halo* mice, 3 sessions, n=100 SC units); **b**, Peristimulus time histograms (PSTHs) for laser OFF (black) vs. laser ON (orange). **c**, Spontaneous activity (500-ms window before stimulus onset), **d,** Sensory-evoked firing rates (100-ms window from stimulus onset) for whisker-responsive neurons at stimulus amplitude 10 (n=28 neurons) and **e**, same as d but baseline-subtracted firing rates; **f,** Population histogram of optogenetic modulation indices (OMI) for spontaneous activity and **g**, stimulus evoked activity. **h-n,** same as a-g but S1 recording during SC inactivation (1 *Pitx2-Halo* mouse, 2 sessions; n=70 S1 units). Error bars: mean ± s.e.m.; f, g, m, n: units with significant modulation are indicated in solid orange colors (p<0.05, paired t-test). NS, not significant; ND, not defined (units that had no spikes during analysis window), not included in histogram, but shown in pie charts.

To determine the role of SC during tactile detection, we optogenetically inactivated the intermediate layers of central SC, contralateral to the whisker stimulus (SC_contra_), where S1 neurons directly project, and C2 whisker-responsive neurons reside (Extended Data Fig. 3c, Extended Data Fig. 7b, d). We used *Pitx2-Cre* knock-in mice to drive stable expression of halorhodopsin (*Pitx2-Halo*), which selectively expresses inhibitory halorhodopsin in around half of excitatory neurons in the intermediate SC^38^ (Fig. 2c, *Pitx2-GFP*). *Pitx2-Cre* expressing neurons include a subset of neurons that receive direct input from S1 as well as the trigeminal brainstem nuclei^39^, and have been shown to represent premotor neurons that mediate head-orienting movements^38^. We first validated functional silencing in *Pitx2-Halo* mice by recording from the C2 whisker-responsive region of SC. Optogenetic inactivation of *Pitx2-Halo* neurons partially reduced both spontaneous firing rates (laser OFF, 6.06 ± 0.89 Hz; ON, 4.22 ± 0.83 Hz, *p* = *6.2*×*10*^*−4*^) and whisker-evoked response amplitudes (Extended Data Fig. 4a–g, OFF, 13.44 ± 3.39; ON, 10.51 ± 3.05, *p = 0.02*). We next determined the effect of SC inactivation on behavioral performance. Despite limited overall suppression of SC activity, photoinhibition of SC_contra_ in *Pitx2-Halo* mice produced strong and consistent increases in detection threshold and decreased probability of ‘yes’ response (ipsilateral / right licks) across all amplitudes, leading to an increase in bias (Fig 2. d–g), strikingly similar to the effects of S1_contra_ inactivation (Fig. 1j).

Recent studies suggest that SC can influence S1 indirectly via the higher-order somatosensory thalamus (posteromedial thalamus, POm)^40^, which in turn projects to S1 (Fig. 2b). This raised the possibility that *Pitx2-Halo* mediated SC inactivation might indirectly suppress S1 activity, accounting for the similar effects of S1_contra_ and SC_contra_ inactivation on behavior. To test this, we recorded across cortical layers of the C2 barrel in S1_contra_ during SC_contra_ inactivation (Extended Data Fig. 4h–n). We did not detect significant changes in S1_contra_ with SC inactivation, suggesting that the behavioral effects of SC_contra_ inactivation in *Pitx2-Halo* mice were unlikely to be due to indirect suppression of S1.

### Unilateral SC inactivation bidirectionally shifts lateralized choice bias

Recent studies across primates and rodents have shown that unilateral perturbation of SC can directionally bias perceptual decisions: increasing SC activity biases attention and choices towards the contralateral side, while decreasing SC activity biases decisions towards stimuli presented ipsilaterally^20,23,41,42^. These effects are thought to stem from competitive dynamics between the two SC hemispheres, which may exert mutual inhibition to mediate lateralized choice selection^21,24,43,44^.

To determine whether SC inactivation can bidirectionally modulate choice bias during our tactile detection task, we next inactivated SC ipsilateral to the whisker stimulus (SC_ipsi,_ Fig. 2i). Strikingly, SC_ipsi_ inactivation had the opposite effect from SC_contra_ inactivation, significantly decreasing the detection threshold (Fig. 2j–k) due to an overall increase in ‘yes’ responses (contralateral licks) across all stimulus amplitudes (Fig. 2j). Thus, unilateral SC inactivation shifts choice bias in opposite directions depending on the targeted hemisphere.

The opposing effects of SC_contra_ and SC_ipsi_ inactivation suggested that the relative activity between the two SC hemispheres can bias choice. Consistent with this notion, bilateral SC inactivation had no significant effect on behavioral performance, including overall detection threshold and bias (Fig. 2n–q, *p = 0.35, 0.80*; paired t-tests). No significant changes in sensitivity were observed across all SC inactivation experiments (Fig. 2h, m, r).

**Extended Data Fig. 5 |:**
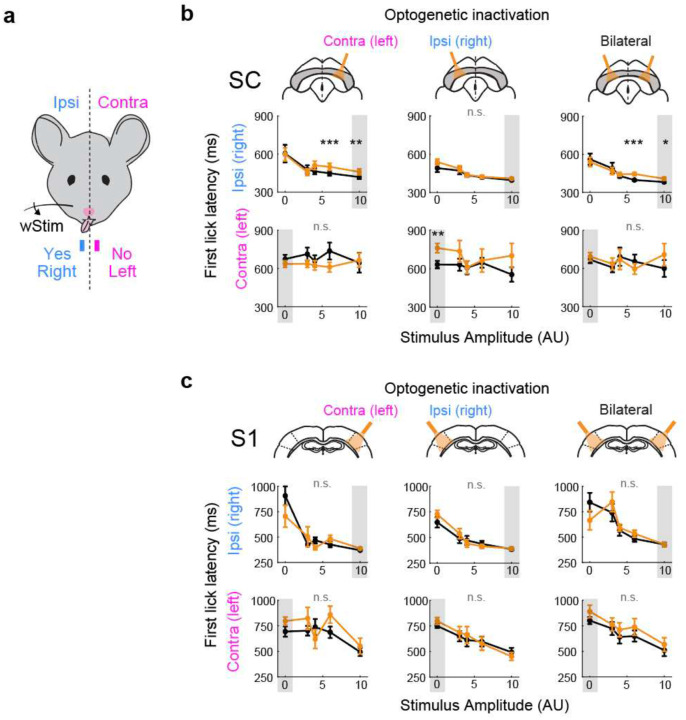
Effects of unilateral and bilateral S1 or SC inactivation on lick response timing. **a.** Schematic of the forced-choice, yes/no tactile detection task. Mice reported stimulus detection by licking right (‘yes’) and stimulus absence by licking left (‘no’). Ipsilateral and contralateral side are defined relative the whisker stimulus. Animal’s right (ipsi, in cyan) and left (contra, in magenta) sides are indicated. **b,** Average lick latencies for the same experiments shown in Fig. 2, SC inactivation in *Pitx2-Halo* mice in contralateral (left), ipsilateral (right), or bilateral SC (contra/ipsi is defined relative to the whisker stimulus). **c,** Same as in b, but for *Emx1-Halo* mice with contra, ipsi, or bilateralS1 inactivation from experiments in Fig. 3. Shaded boxes indicate high-confidence responses (ipsi/right licks for amplitude 10, contra/left licks for amp 0); NS indicates p > 0.05 for all for OFF vs ON values across all amplitudes, * p < 0.05, ** p<0.01, ***p<0.001, n.s., no significant pairs (p>0.05), paired t-tests with Bonferroni correction.

SC-driven bias can reflect distinct processes across tasks and subregion. In particular, inactivation of the lateral SC region has been shown to inhibit contralateral licking, while medial SC does not^21^. Given that the C2 whisker-responsive region targeted for inactivation lies proximal to the lateral SC region that has been implicated in premotor control of licking^21,43,45^, we also considered whether the behavioral bias effects we observed during unilateral SC inactivation were due to a general impairment in direction-specific licking. Unilateral SC inactivation induced stimulus-dependent changes in lick latency, with changes mostly limited to high-confidence trials (amp10 yes/right licks, and amp0 no/left licks), where the interpretation was least confounded by perceptual uncertainty at near-threshold stimuli (Extended Data Fig. 5a, b, gray shaded boxes). Bilateral SC inactivation, despite its minimal impact on behavioral performance (Fig. 2, n–r), induced moderate increases in lick latencies for yes/right responses to the strongest stimuli, with no detectable changes at weaker amplitudes or for no/left licks across any amplitude.

Thus, unilateral SC inactivation in *Pitx2-Halo* mice produced lateralized choice bias, whereas bilateral SC inactivation neutralized this effect. The changes in lick latencies suggest that the choice bias during direct SC-perturbation is at least in part due to a motor bias, consistent with the known role of SC in mediating lateralized licking movements^21^. However, the absence of changes in lick latency across amplitudes argues against a global motor impairment. Taken together, these findings are consistent with models^24,44^ in which choice bias is shaped by relative activity across SC hemispheres rather than by absolute activity within either SC hemisphere alone.

### S1 activity shapes the direction of choice bias

**Fig. 3 |:**
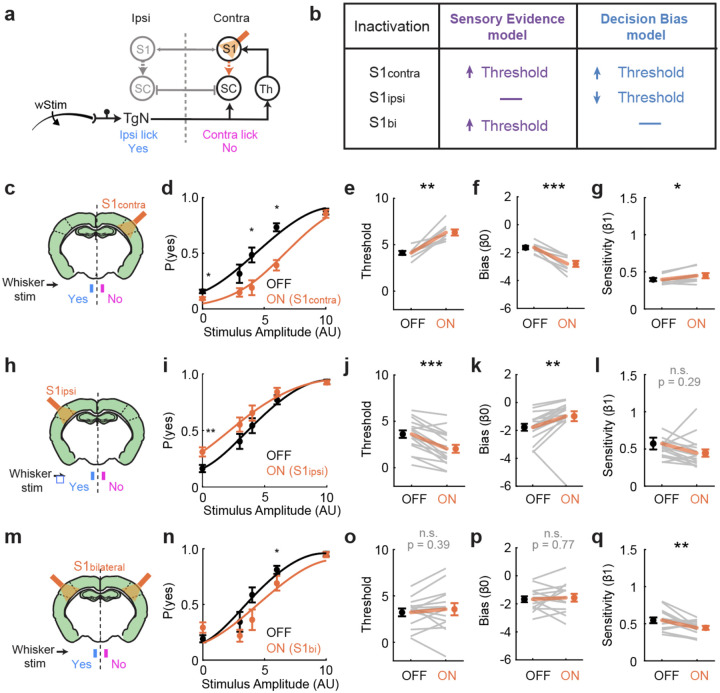
S1 inactivation induces directional shifts in decision bias. **a.** Simplified schematic of S1 and SC pathways for tactile detection. A whisker stimulus (wStim) activates the trigeminal brainstem nuclei (TgN), which send ascending inputs to contralateral S1 via thalamus (Th). S1 projects directly and indirectly to SC in the same hemisphere (dashed orange arrow). **b,** Conceptual models for S1’s primary role in detection behavior: *Sensory* evidence *model*: S1 encodes critical sensory information needed for detection. *Decision bias model*: S1 modulates downstream SC to bias decisions, rather than acting solely as a sensory relay. Predicted outcomes for threshold with inactivation are shown. Arrows indicate predicted increase or decrease; dashes suggest minimal change (contra-, ipsi-, or bilateral relative to whisker stimulus). **c-g,** Experimental results showing outcome of S1_contra_ inactivation on behavior (n=3 mice, 8 sessions). **d,** Probability of ‘yes’ response by stimulus amplitude. **e**, detection threshold; **f,** bias; and **g,** sensitivity. **h-l,** Same as in c-g, but for ipsilateral S1 inactivation (n=6 mice, 17 sessions); and **m-q**, bilateral S1 inactivation (n=6 mice, 14 sessions). Error bars: mean ± s.e.m.; P(yes) and threshold: two-sided Wilcoxon signed-rank test (Bonferroni correction for d, l, n).

A common assumption is that inactivating S1 impairs detection behavior primarily by reducing the amount of sensory information in S1. However, S1 projects directly^29^ (Fig. 3a, Extended Data Fig. 3) and indirectly (via other cortical and basal ganglia pathways) to SC, and SC itself is known to robustly respond to tactile stimuli^15,21,46,47^. The striking similarity between behavioral effects of S1_contra_ and SC_contra_ inactivation led us to consider whether S1_contra_ inactivation impairs detection behavior primarily by modulating downstream SC activity to shift choice bias, rather than simply by disrupting sensory encoding within S1.

To test this hypothesis, we considered two competing models of S1’s role in tactile detection: a *sensory evidence model*, in which S1’s primary role is to encode tactile stimuli during the detection task (Fig. 3b, purple), or a *choice bias model*, in which S1 primarily shapes choice bias during the task (Fig. 3b, blue). Ascending whisker inputs to S1 are lateralized, primarily originating from the contralateral whiskers^30,48–51^. Although ipsilateral whisker responses are present in S1_contra_, these have been shown to originate from cross-callosal inputs from the opposite S1 hemisphere rather than uncrossed ascending input^50,51^. Thus, in the *sensory evidence model*, inactivation of S1 ipsilateral to the whisker stimulus (S1i_psi_) should have minimal behavioral impact. Furthermore, bilateral inactivation should impair detection at least as strongly as S1_contra_ inactivation, due to the loss of sensory responses in both hemispheres. In contrast, under the *decision bias model*, S1 influences detection behavior primarily through lateralized downstream effects on decision circuits such as SC. This model predicts that S1_ipsi_ and bilateral S1 inactivation should mirror those seen with equivalent SC manipulations: S1_ipsi_ should decrease detection threshold due to choice bias, and bilateral inactivation should neutralize bias, producing minimal effects on threshold (Fig. 3b).

To directly test these model predictions, we trained a new cohort of *Emx1-Halo* mice and inactivated either contra-, ipsi-, or bilateral S1 in each session. S1_contra_ inactivation increased detection threshold (Fig. 3e, *p = 7.0e-4*) and conservative bias (Fig. 3f, *p = 3.2e-4*), consistent with the original experiments in Fig. 1. We observed a modest increase in sensitivity at the session level (Fig. 3g; n=8 sessions, 3 mice, p = 0.02), but this did not replicate in the larger cohort (Fig. 1k; n=22 sessions, 6 mice, *p = 0.09*). In contrast, threshold and bias shifts were reproducible and consistent across animals and sessions (Fig. 3e, f).

We next tested the effects of S1_ipsi_ inactivation, which surprisingly led to a consistent *decrease* in the detection threshold and liberal bias shift, with animals more likely to say ‘yes’ across stimuli--even during no-stimulus trials (Fig. 3h–k; n=6 mice, 17 sessions). The decreased detection threshold was driven by an increase in bias (Fig. 3k), rather than due to enhanced sensitivity (Fig. 3l).

Bilateral S1 inactivation did not significantly alter detection threshold or decision bias (Fig. 3o, p; *p = 0.39, p = 0.77*; n=6 mice, 14 sessions), but induced a modest decrease in detection probability for near-threshold amplitude 6 (Fig. 3n, *p = 0.04*, Wilcoxon signed-rank test with Bonferroni correction), and revealed a small but significant decrease in sensitivity (Fig. 3q, *p = 0.009*), suggesting that S1 contributes to enhancing sensory processing near threshold, but this role may have been masked by the stronger effects of bias shifts induced by unilateral S1 inactivation.

Together, these results indicate that behavioral impairments during unilateral S1 inactivation are consistent with lateralized modulation of downstream circuits that shift bias, rather than by loss of sensory information in S1—more closely aligned with the *decision bias model* than *sensory evidence model* (Fig. 3b). Overall, these findings support a model in which S1 primarily shapes decision bias during tactile detection behavior, while making sensory contributions for near-threshold stimuli.

**Extended Data Fig. 6 |:**
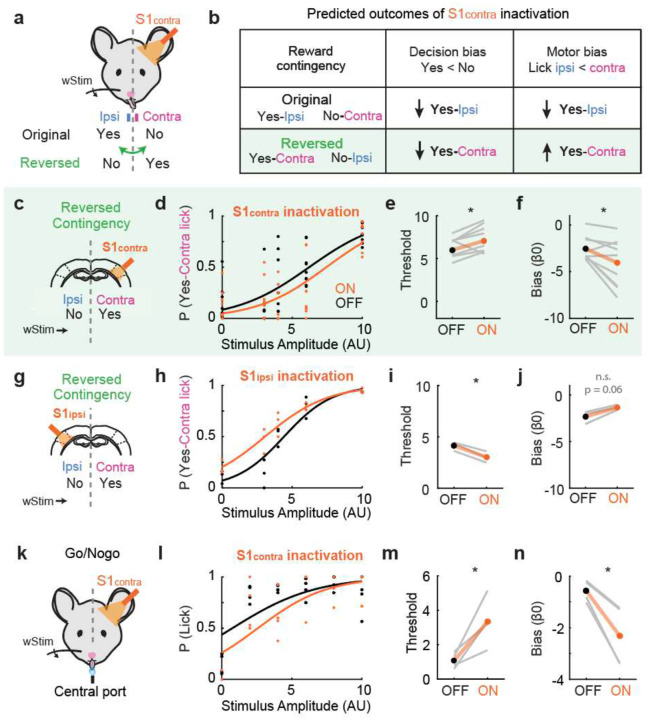
S1 inactivation induces a decision bias rather than motor bias. **a,** Original paradigm: S1 inactivation decreased ‘yes’ (ipsilateral lick) responses, which could be due to a perceptual bias or a motor bias. To distinguish between perceptual vs. motor bias, we trained mice on the reverse reward contingency; subjects were trained to lick contralateral to the whisker stimulus for ‘yes’ (yes-contra) or ipsilaterally for a ‘no’ response--opposite of the original paradigm. **b,** Expected outcomes for S1_contra_ inactivation-induced perceptual bias vs. motor bias outcomes are shown. Under the reversed contingency motor mapping, **c,** S1_contra_ inactivation leads to **d,** decreased tendency to respond ‘yes-contra’; **e,** increased detection thresholds and **f,** conservative bias (n=3 mice, 9 sessions). **g,** S1_ipsi_ inactivation leads to **h,** an increase in ‘yes-contra’ responses; **i**, decreased threshold and **j,** liberal bias (n=1 mouse; 3sessions). **k,** S1_contra_ inactivation in animals trained on a Go/Nogo version of the task with a single, central port. S1_contra_ inactivation **l**, decreased the probability of detection (P(Lick)); **m,** increased detection threshold and; **n,** decreased decision bias, despite no requirement for directional licking movements (n=2 mice, 5 sessions). These results suggests that bias shifts during S1 inactivation is due to a perceptual, rather than motor bias. Error bars: mean ± s.e.m.; paired t-tests.

S1 projects to multiple downstream motor-related areas^11,52^ (e.g., primary and secondary motor cortices, SC, and basal ganglia), raising the possibility that the effects of unilateral S1 inactivation reflect a lateralized motor bias, rather than perceptual decision bias. To test this, we trained a new cohort of mice on a reversed-contingency task in which the motor mapping of Yes/No choices was swapped, allowing us to dissociate sensory-driven perceptual bias from lateralized motor bias. In the original paradigm, ‘yes’ was mapped to licking ipsilaterally towards the whisker stimulus, and S1_contra_ inactivation led to decreased ‘yes’ and ipsilateral lick responses. In the reversed motor contingency paradigm, animals were required to lick contralaterally, away from the same stimulus to respond ‘yes’ (Extended Data Fig. 6a, b). S1 inactivation under the reversed contingency still decreased ‘yes’ responses despite the opposite lick-direction mapping, consistent with a perceptual bias rather than directional motor bias (Extended Data Fig. 6c–f). Similarly, S1_ipsi_ inactivation during the reversed motor contingency paradigm *increased* ‘yes’ responses and contra-licking, consistent with a perceptual decision (Yes/No) rather than motor (left/right) bias.

No significant differences were detected for sensitivity (S1_contra_ inactivation, *p=0.10*; S1_ipsi_ inactivation, *p=0.26*, paired t-test, data not shown). Moreover, we did not detect significant changes in lick latencies with unilateral or bilateral S1 inactivation, further suggesting that S1 inactivation-induced choice bias was not simply due to a motor bias (Extended Data Fig. 5c).

To further rule out lateralized motor impairments, we tested a new group of animals on a Go/Nogo version of the task, in which detection responses were made by licking a single, central lick port, eliminating lateralized motor requirements. Even in this task, S1_contra_ inactivation increased detection threshold driven by a conservative bias shift, mirroring the Yes/No tasks (Extended Data Fig. 6k–n). Together, these results suggest that S1 modulates perceptual decision bias, rather than simply biasing lick direction.

### Stimulus encoding is greater in S1 than SC, but choice prediction is similar across areas

**Extended Data Fig. 7 |:**
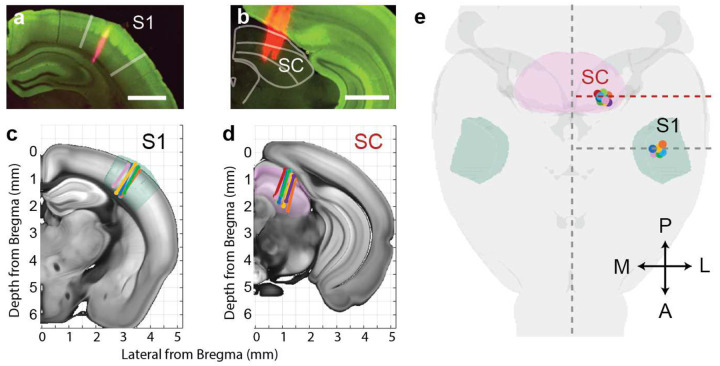
Recording locations for C2-whisker responsive areas in S1 and SC. **a,** Example histology image showing dye-coated probe tracts in S1 and **b**, SC in an *Emx1-Halo* mouse (n=1). Confirmed probe locations overlaid to a coronal atlas reference from the Neuropixels trajectory explorer software showing targeting relative to Bregma (30° insertion angle, AP −1.6, ML 3.5, ventral −1.3-mm) for **c,** S1; and **d,** SC (20° insertion angle; AP −3.7, ML 1.7, ventral: −2.5). **e**, Top-down view of reconstructed probe locations using the AP-histology software, overlaid with a mouse brain image from the Neuropixels trajectory explorer software. n= 6 mice. Scale bar in a, b: 1mm.

**Fig. 4 |:**
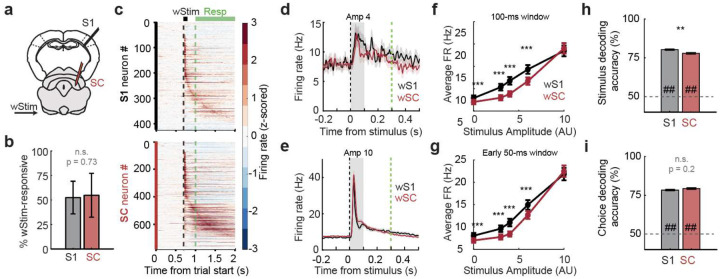
Sensory-evoked responses in S1 vs. SC during tactile detection behavior. **a,** Simultaneous array recordings in S1 and SC contralateral to the whisker stimulus (wStim) during the detection task. **b,** Similar proportions of neurons responded during the stimulus window (100ms from stimulus onset); p=0.73, unpaired t-test; n=17 sessions, 6 mice. **c,** Z-scored firing rates for each single unit to stimulus (amp 10), sorted by peak firing time. Top: S1 (n=423 neurons); bottom: SC (n=778 neurons, simultaneous S1 and SC recordings in 6 mice, 17 sessions, laser-OFF trials only). **d,** Average PSTHs of whisker-responsive neurons in S1 (wS1, black, n=209 neurons) and SC (wSC, red, n=412 neurons) for stimulus amp 4 and **e,** amp 10; **f,** Average firing rates of whisker stimulus-responsive S1 (wS1, black) and SC (wSC, red) units during the stimulus window (100-ms from stimulus onset, shaded gray in d and e), Wilcoxon rank-sum test with Bonferroni correction; **g,** Same as in f, but for normalized (baseline-subtracted) firing rates; **h,** Stimulus decoding (amp 0 vs. 6) and **i,** choice decoding (yes vs. no response at amp 6) accuracy during the stimulus window. ## p<0.01 indicates significant difference from 50% chance (shuffle-label null test); S1 vs. SC, paired t-test per resample run (n=282 units matched across areas and conditions). Black dashed lines indicate stimulus onset, green dashed lines indicate start of lick response window (300-ms from stimulus onset). Error bars: mean ± s.e.m.

SC receives direct bottom-up tactile inputs from the trigeminal brainstem nuclei, as well as top-down inputs from S1 (Extended Data Fig. 3). The behavioral similarity between S1 and SC inactivation suggested that S1 may influence detection behavior by modulating downstream SC activity. While the neural correlates of tactile detection have been extensively characterized in S1, how SC neurons contribute to this process and, further, how S1 and SC may together coordinate detection behavior, remain poorly understood. As a first step, we characterized how whisker stimuli are represented in S1 and SC during detection behavior. We simultaneously recorded from the C2-barrel in S1_contra_ and the somatotopically aligned, C2-responsive area in SC_contra_ during the Yes/No detection task (Fig. 4a, Extended Data Fig. 7; S1: n = 423 neurons; SC: n = 778 neurons; n=6 mice, 17 sessions).

Despite the heterogeneity of neurons in the intermediate SC layers, which include multi-sensory and pre-motor neurons, around half of SC neurons in the targeted recording site responded to C2 whisker deflection, a proportion similar to the C2 barrel in S1 (Fig. 4b; S1, 52.4 ± 16.6%, SC, 54.8 ± 22.4%, p = 0.73, unpaired t-test). Activity in both areas tiled the period from stimulus onset (Fig. 4c, black dashed lines) through the response window (green lines, starting 300 ms after stimulus onset). S1 neurons had significantly higher stimulus-evoked responses for weaker stimuli compared to SC (Fig. 4f, S1, n=216; SC, n=396 whisker stimulus-responsive units). SC neurons have been shown to increase activity during motor action, such as whisking^53^, licking^21,45^, or preparatory motor activity^21,47,54^, which can confound stimulus activity from detection-induced pre-motor responses. We therefore tested a more restricted stimulus window of 50 ms from stimulus onset, which showed similar differences between S1 and SC (Fig. 4g). Thus, average stimulus-evoked firing rates indicate stronger encoding of low-amplitude tactile stimuli in S1 than in SC during detection behavior.

We determined how accurately S1 vs. SC population activity predicted stimulus presence and choice under normal (laser-OFF) conditions. To decode stimulus presence, we trained linear support vector machine (SVM) classifiers to distinguish between no-stimulus (amp 0) and near-threshold stimulus (amp 6) trials during the stimulus window (100 ms from stimulus onset), while balancing trials by lick direction. Both S1 and SC activity robustly predicted stimulus presence, but S1 showed higher accuracy, consistent with stronger stimulus-evoked responses at near-threshold amplitudes in S1 (Fig. 4h, S1, 86.2 ± 0.4%; SC, 81.1 ± 0.5%; n=282 units matched across areas, *p = 0.001, paired t-test*).

We next asked how accurately the population activity during the same stimulus window could predict the animal’s subsequent choice on a trial-by-trial basis. Choice was reliably decoded from both S1 and SC with similar accuracy (Fig. 4i, S1, 78.4 ± 0.5%; SC, 79.3 ± 0.5% n = 282 units, *p = 0.2, paired t-test*). Thus, although S1 contained stronger near-threshold stimulus representation, both S1 and SC population activity were similarly predictive of trial-by-trial choice.

### S1 inactivation decreases spontaneous and stimulus-evoked responses in SC.

**Extended Data Fig. 8 |:**
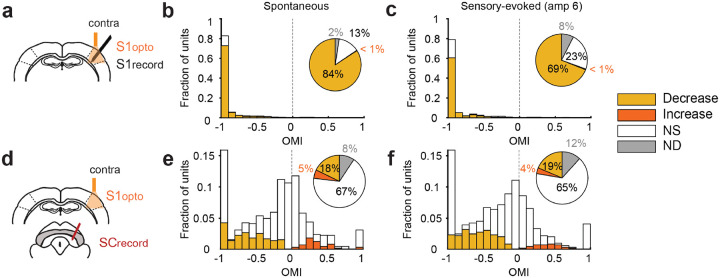
Optogenetic modulation of S1_contra_ and SC _contra_ neurons during S1_contra_ inactivation. **a,** S1_contra_ recordings during optogenetic S1_contra_ inactivation (S1opto). **b,** Optogenetic modulation index (OMI = (ON-OFF)/(ON+OFF)) for spontaneous **c,** stimulus-evoked activity S1_contra_ neurons (near-threshold amplitude 6, n=423 single units). **d-f,** Same as in a-c, but for SC_contra_ (n=778 single units) during S1_contra_ inactivation. Dark orange indicates significantly increased; light orange shows significantly decreased activity (p < 0.05). NS, not significant, ND, not defined, units with low firing rates that did not spike during the analysis window (gray, shown in pie chart); n = 6 mice, 17 sessions.

**Fig. 5 |:**
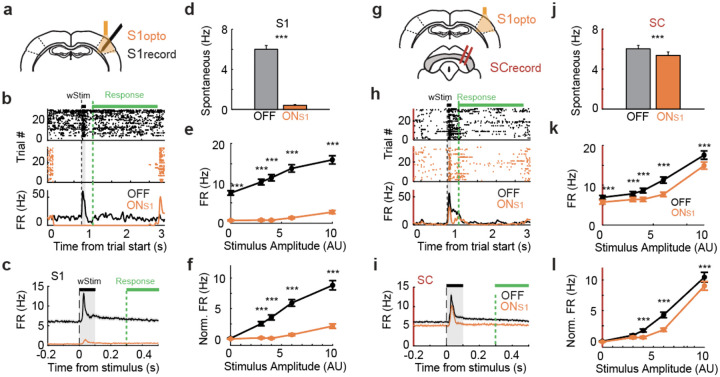
Inactivation of S1 decreases spontaneous and evoked firing rates in SC. **a-f,** S1 and **g-l,** SC recordings during contralateral S1 inactivation (6 mice, 17 sessions)**. a,** schematic showing S1 recordings during S1opto. **b,** Raster plots and PSTHs for an example neuron in S1. **c,** Average PSTH of S1 neurons across laser OFF vs. ON_S1_ trials (n=423 neurons); **d,** S1 spontaneous firing rates (n=423); **e,** Average sensory-evoked firing rates of whisker-responsive neurons (n=216) and **f,** Normalized (baseline-subtracted) firing rates of the same data in e. **g**, Schematic of SC recordings during S1opto. **h-l,** Same as b-f, but for SC neurons (**i, j**: all SC units, n=778; **k, l**: stimulus-responsive SC units n=396). Black dashed lines: stimulus onset; green dashed lines: start of response window; gray shaded boxes: stimulus window used for analyses in e, f, k, l; Error bars: mean ± s.e.m. Paired t-tests, Bonferroni correction for multiple comparisons.

We next asked whether S1_contra_ inactivation impairs behavior due to loss of sensory information in S1, or by disrupting activity downstream in SC_contra_. To test this, we analyzed the laser ON trials during simultaneous recordings from S1_contra_ and SC_contra_. As previously reported^9^, optogenetic inactivation of S1_contra_ in *Emx1-Halo* mice was highly efficacious (Fig. 5a–f), locally suppressing 93% of overall baseline firing rates, with the majority of neurons being significantly modulated (Extended Data Fig. 8a–c). Despite strong inactivation efficacy, some residual activity for high-amplitude stimuli remained (Fig. 5e, f; 15% residual activity for the strongest stimulus, amp 10).

In SC_contra_, S1_contra_ inactivation produced heterogeneous effects across single units, with some significantly decreasing activity (18–19% of single units), and a small portion showing moderate increases (4–5% of single units, Extended Data Fig. 8d–f). Overall, there was a significant decrease in average spontaneous and stimulus-evoked activity across all stimulus amplitudes tested (Fig. 5j–l). Thus, S1_contra_ inactivation significantly decreased the baseline firing rates as well as sensory-evoked responses in SC_contra_, potentially impairing SC’s ability to mediate tactile detection behavior beyond its local effects in S1_contra_.

### Changes in SC, rather than S1 population activity, predict choice during S1 inactivation

**Fig. 6 |:**
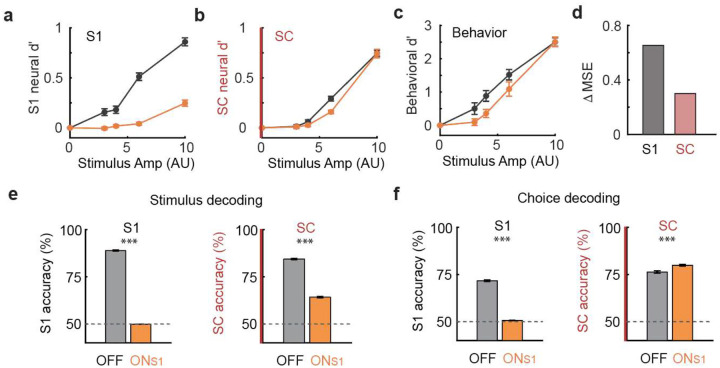
Behavioral changes with S1 inactivation are better captured by SC than S1activity. **a,** Discriminability (d’) for no-stimulus (amp 0) vs. each stimulus amplitude, shown for stimulus-responsive neurons in S1, **b,** SC; and for **c,** behavior under control (laser OFF, black) vs. S1_contra_ inactivation (ON, orange). **d,** Mean squared error (ΔMSE) between min-max normalized Δd’ curves (OFF-ON) from neural data (S1, SC) and behavior across non-zero amplitudes; lower ΔMSE indicates a closer match in the pattern of inactivation-induced change. **e,** Stimulus decoding accuracy (amp 0 vs. amp 6) from S1 and SC population activity during the stimulus window (100ms from stimulus onset), using a linear SVM classifier trained on OFF trials, tested on OFF vs. ON trials. **f,** Choice decoding accuracy (yes/no at near-threshold amp 6) for S1 and SC. Neural and behavioral d’ were computed from AUROC measurements comparing stimulus vs. no-stimulus trials (see Methods). Horizontal dashed lines: chance level; #: p<0.05, ## p<0.01 indicates significant difference from chance (shuffle-label null test); S1 vs. SC: *** p< 0.001, paired t-test per resample run. Error bars: mean ± s.e.m., paired t-test.

Although photoinhibition of S1_contra_ substantially reduced S1_contra_ activity (Fig. 5b–f, Extended Data Fig. 8a–c), prior work suggests only a small amount of S1 activity may be needed to support stimulus detection^55,56^. We next asked whether decreased sensory-evoked responses in S1 or SC were more correlated with behavioral impairments observed during S1_contra_ inactivation. To compare neural and behavioral effects, we quantified d’ (d-prime) across stimulus amplitudes for S1 and SC neural activity and behavioral choices. Consistent with efficient optogenetic silencing, S1_contra_ neural d’ during S1_contra_ inactivation (laser-ON trials) hovered around 0, with some residual discriminability at stronger amplitudes (Fig. 6a). In SC_contra_, S1_contra_ inactivation decreased neural d’ only for near-threshold stimulus amplitudes (Fig. 6b). Similarly, S1 inactivation decreased behavioral d’ only at near-threshold amplitudes (Fig. 6c). To compare how well S1 vs. SC captured the change in behavioral d’, we computed the difference in mean-squared error (ΔMSE) between neural and behavioral d’ (ON-OFF) profiles across stimulus amplitudes (Methods). A lower ΔMSE indicates a closer match in the pattern of inactivation-induced changes between neural and behavioral d’ curves, independent of absolute scale and offset. The ΔMSE was substantially lower for SC than S1 (Fig. 6d, S1: 0.65 vs. SC: 0.30), indicating that behavioral changes with S1 inactivation more closely mirrored changes in SC activity than S1.

To extend these analyses, we tested whether sensory-evoked activity in S1 or SC could reliably predict stimulus and behavioral choice on a trial-by-trial basis. We applied cross-condition decoding analyses using SVM classifiers trained on OFF trials, and tested their performance on held-out OFF or ON trials. We restricted our analysis to the stimulus window (100-ms from stimulus onset for near-threshold amp 6) before licking or reward delivery. To further control for potential motor confounds, we matched trials by lick direction. Stimulus decoding accuracy dropped to chance-level in S1 during S1 inactivation (ON), and was significantly reduced in SC (Fig. 6e, S1, OFF: 88.9 ± 0.35 vs. ON_S1_: 49.9 ± 0.08, p < 0.001; SC, OFF: 84.4 ± 0.42, ON_S1_: 64.2 ± 0.46 %, p<0.001, paired t-test).

If residual S1 activity is responsible for the remaining task performance during S1 inactivation, then an OFF-trained S1 decoder should continue to predict the subject’s altered choice patterns on laser-ON trials. Instead, S1 choice decoding accuracy dropped to chance for laser-ON trials (Fig. 6f, S1, OFF: 71.7 ± 0.18% vs. ON_S1_: 50.66 ± 0.18%, p < 0.001, paired t-test). In contrast, SC choice decoding accuracy remained robust across conditions, even showing a modest increase (Fig. 6f, SC, OFF: 76.3 ± 0.64, ON_S1_: 79.89± 0.50 %, p<0.001), suggesting that SC population activity during the stimulus window continued to predict the subject’s choices on a trial-by-trial basis.

Together, these results suggest that behavioral impairments during S1 inactivation were more closely associated with changes in SC population activity during the stimulus window than with loss of S1 activity. S1 inactivation disrupted stimulus representations in both S1 and SC, but only SC activity reliably tracked behavioral choice across conditions, supporting a model in which SC, rather than residual activity in S1, better explains shifts in behavioral impairments observed during S1_contra_ inactivation.

### S1 inactivation-induced changes in behavior are SC-dependent

**Extended Data Fig. 9 |:**
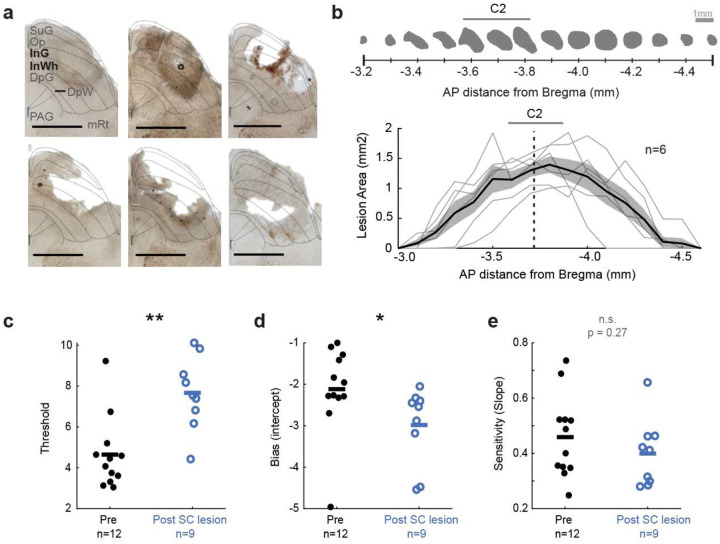
SC lesions and performance across sessions. **a,** Representative histology sections for SC_contra_ lesions in 6 animals included in the study. Paxinos atlas contours were overlaid to approximate SC layers. Lesions were targeted to the intermediate SC layers (InG and InWh), where C2-whisker responsive neurons were located, while maximally avoiding deep and lateral SC motor layers (DpG, DpW). Lesions that extended into the PAG or mRt were excluded. **b,** Top: Shape of the SC across anterior-posterior histology sections. Location of C2-whisker responsive recordings identified by dye labeled probe tracks are indicated. Bottom: Quantification of lesion area across 100um histology sections and approximate AP location from Bregma. **c-e,** Effects of SC lesion on detection performance, pooled session data: **c,** Detection threshold; **d,** decision bias (intercept); **e,** sensitivity (slope), before (black) and after SC lesions (blue); circles represent individual sessions. Only sessions with reliable logistic fits (see Methods) were included in these analyses, resulting in variable sample sizes across metrics. Two-sided Wilcoxon rank-sum test. **f,** Percent correct performance (includes all sessions, n=6 mice), **i**, bias (intercept); and **j,** sensitivity (slope). Bias and sensitivity are logistic regression-derived parameters; only sessions with reliable logistic fits were included, resulting in variable sample sizes across sessions. Orange dots and lines represent S1_contra_ inactivation (laser ON) conditions, black is laser OFF. Two-sided Wilcoxon signed-rank test within session (OFF vs. ON), with Bonferroni correction for multiple comparisons. NS: not significant. Abbrev. Superior colliculus (SC) laminae: SuG, superficial gray layer of SC; Op, optic nerve layer; InG, intermediate gray layer; InWh, intermediate white layer; DpG, deep gray layer; DpW, deep white layer. PAG, periaqueductal gray; mRt, medial reticular nucleus. Scale bars: 1mm. Error bars: mean and s.e.m.

**Fig. 7 |:**
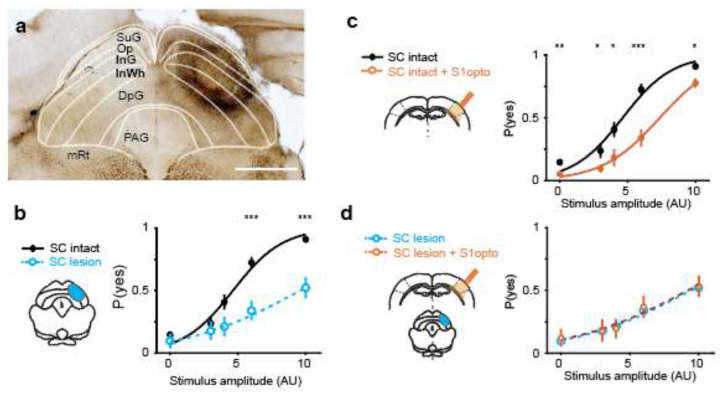
S1 inactivation has minimal behavioral effects in the absence of SC. **a,** Brightfield image of an example SC lesion. Lesions were targeted via laser ablation in contralateral SC. **b,** Probability of ‘yes’ response (P(yes) before (solid black line, filled circles, average of 2 sessions pre-SC lesion) and after (cyan, dashed line, open circles, 2 sessions post-SC lesion) contralateral SC lesions. **c,** Pre-SC lesion: baseline performance (black, same as in b) vs. contra-S1 inactivation (S1 opto, orange) in the intact animal. **d,** Post-SC lesion: baseline performance with SC lesion (cyan, same as in b), and S1 inactivation in SC lesioned animals (orange, dashed lines). Dots represent individual animals. Statistical comparisons between conditions were performed using a linear mixed-effects model including the condition as a fixed effect and animal as a random effect; p-values were Bonferroni corrected across amplitudes; n = 6 mice. Scalebar: 1mm.

S1 projects to several downstream targets, and prior work has shown that chemogenetic silencing of S1 axon terminals in either SC, striatum, or POm impaired detection behavior^11^. While our data revealed a strong correlation between changes in SC population activity and behavioral choice, the effects of S1 inactivation could be mediated by alternative pathways independent of the SC. To directly test whether the behavioral changes during S1 inactivation is SC-dependent, we combined S1 inactivation with SC lesions. SC_contra_ lesions were performed by laser ablation targeted to the C2 whisker-responsive region (Fig. 7a, Extended Data Fig. 9a). Lesions were calibrated to target the intermediate layers of SC while avoiding lateral SC (Extended Data Fig. 9a, b; lesion diameter: 1.6 ± 0.14 mm; n = 6 mice).

We first assessed the behavioral effect of SC lesions alone. SC_contra_ lesions strongly impaired detection performance, increasing detection thresholds (Fig. 7b, Extended Data Fig. 9). Notably, SC lesions produced stronger impairments compared to *Pitx2-Halo* mediated SC_contra_ optogenetic suppression, due to the larger extent of the lesion that spanned the superficial and intermediate layers (Extended Data Fig. 9a). In a few sessions, SC lesions resulted in strong impairments, leading to unstable psychometric fits, limiting reliable interpretation of bias and sensitivity parameters. Despite this, analysis of the remaining sessions showed significantly increased thresholds and a conservative shift in choice bias. No significant changes in sensitivity were detected (Extended Data Fig. 9).

While animals were significantly impaired after SC lesions, they retained some ability to perform the detection task at strong stimulus amplitudes (Fig. 7b), which could be mediated by the intact S1 pathway^15^. We next asked how S1 inactivation influences detection in the absence of SC. As a baseline, we confirmed that S1_contra_ inactivation reliably reduced detection probability prior to SC_contra_ lesions in this cohort (Fig. 7c), consistent with earlier results. Strikingly, following SC lesions, additional inactivation of S1 did not further impair detection performance (Fig. 7d). Together, these results suggest that behavioral effects of S1 inactivation depend on SC activity, suggesting that SC serves as a key downstream pathway through which S1 modulates detection behavior.

Taken together, these results demonstrate that the behavioral impairment associated with S1 inactivation is primarily due to a shift in perceptual decision bias, which is dependent on SC activity. Our results support a model in S1 contributes to tactile detection not only by locally encoding sensory information, but by enhancing sensory-evoked responses in SC, and modulating SC-dependent circuits that determine how tactile evidence is converted into perceptual decisions.

## DISCUSSION

A common interpretation is that S1 inactivation impairs detection behavior due to the loss of sensory information encoded locally in S1. This is a reasonable view given that contralateral S1 is the primary cortical target for somatosensory input, and numerous studies have shown that inactivation of contralateral S1 increases the detection threshold for whisker stimuli^15,4,10,5,12,9,13^. However, several key results argue against a simple loss-of-sensory-information model. First, although contralateral S1 inactivation decreased detection probability as expected, ipsilateral S1 inactivation produced the opposite effect. Second, these opposite effects on detection probability also extended to no-stimulus trials, significantly decreasing or increasing false alarm rates depending on the hemisphere inactivated. Third, bilateral S1 inactivation led to smaller changes in detection threshold than either contra- or ipsilateral inactivation alone. Fourth, psychometric analysis revealed that these behavioral effects of S1 inactivation were more consistent with directional shifts in choice bias, than with changes in sensitivity (Fig. 3). Finally, S1 inactivation decreased spontaneous and sensory-evoked neural activity in SC, and trial-by-trial choice was more reliably predictable from the changes in population activity in SC, rather than decreased sensory responses in S1 (Fig. 6f). Thus, the behavioral effects of S1 inactivation cannot be explained simply by decreased sensory evidence encoded in S1.

An alternative possibility is that S1 and SC function as parallel sensory pathways with different neurometric thresholds. In this model, S1 serves as the default, low-threshold pathway, but when S1 is inactivated, the SC supports residual, higher threshold detection behavior. Consistent with this view, S1 responds to weak tactile stimuli more reliably than SC (Fig. 4 f–h), and prior work suggests that in the absence of S1, SC can support detection of salient, but not weak stimuli^15^. However, our results argue against a dual threshold model. First, SC lesions impaired detection behavior even when S1 remained intact^15^ (Fig. 7b). Second, in the absence of SC activity, additional S1 inactivation did not impair the residual detection performance (Fig. 7d). Thus, although S1 and SC both carry tactile stimulus information, the behavioral effects of S1 inactivation are more consistent with a lateralized shift in decision bias via changes in SC activity, rather than the unmasking of a backup SC pathway when S1 activity is lost.

What is the role of S1 and SC during detection behavior? Our results are consistent with a model in which S1 contributes to tactile detection not only by enhancing sensitivity for weak stimuli, but also by modulating SC to shape action selection. At the neuronal level, S1_contra_ inactivation decreased spontaneous and sensory-evoked activity in downstream SC_contra_, potentially reducing the likelihood that sensory input reaches the threshold for a detection response.

How do changes in SC activity during S1 inactivation lead to choice bias? S1 inactivation-induced choice bias was more consistent with a perceptual bias rather than a motor bias. The reversed motor contingency experiments (Extended Data Fig. 6) suggest that unilateral S1 inactivation biased perceptual decisions (Yes/No) independent of motor action (lick right/left). In contrast, unilateral inactivation of lateral SC is known to directly impair directional licking output^21,45^, consistent with the strong directional bias we observed with *Pitx2-Halo* mediated SC inactivation (Fig. 2). Given the current data, we cannot rule out whether S1 inactivation also alters premotor/motor output neurons outside of the recording site in lateral SC. Notably, whisker S1 axons project not only to the stimulus-responsive neurons in the upper region of the intermediate layers, but also the lower intermediate and deep SC layers where premotor/output neurons reside (Extended Data Fig. 3). One possibility is that, in addition to suppressing stimulus-evoked responses in SC, S1 inactivation also directly or indirectly affects the premotor/output neurons to bias action selection. Thus, although the dominant effect of S1 inactivation was a shift in perceptual bias, we cannot rule out a weaker motor bias effect that may have been masked by the larger perceptual bias observed during unilateral S1 inactivation.

Although *Pitx2-Halo*-mediated SC inactivation experiments produced strong shifts in choice bias, we did not observe changes in sensitivity. Given the non-overlapping expression pattern of Pitx2-Halo and S1 projection areas noted above, *Pitx2-Halo* likely labels only a subset of excitatory neurons in the intermediate SC, excluding a large portion of tactile sensory SC neurons. Contralateral SC lesions did not reveal in clear changes in sensitivity, although these effects were difficult to interpret given the severe behavioral impairment and stronger effects on choice bias. Future experiments targeting SC neurons that receive ascending trigeminal brainstem sensory input would help determine whether distinct SC neurons contribute separately to tactile sensitivity and decision bias.

In a Go/Nogo detection task that does not require lateralized motor output, S1_contra_ inactivation also altered choice bias rather than sensitivity (Extended Data Fig. 6 k–n). An interesting prediction is that ipsilateral S1 inactivation would also bias detection behavior in the Go/Nogo paradigm. Future experiments are needed to test this possibility.

**Extended Data Fig. 10 |:**
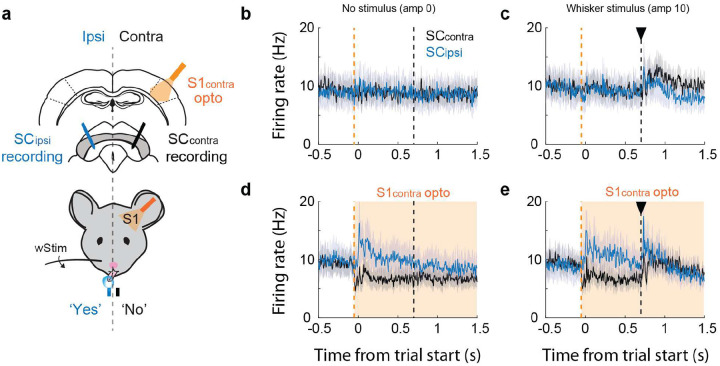
Unilateral S1 inactivation induces an interhemispheric imbalance in SC activity. **a,** Schematic of experiment. Ipsilateral (ipsi) and contralateral (contra) sides are defined relative to the whisker stimulus. S1 contralateral to the whisker stimulus (S1_contra_) was optogenetically inactivated during simultaneous bilateral recording from ipsilateral (SC_ipsi_) and contralateral SC (SC_contra_) during the yes/no detection task. **b, c**, Average firing rates during laser-OFF trials for **b,** no-stimulus trials and **c**, high-amplitude whisker stimulus trials. **d, e,** Mean firing rates during S1_contra_-inactivation trials (S1_contra_ opto) for **d,** no-stimulus trials and **e,** high-amplitude stimulus trials. During S1 inactivation, activity decreased in SC_contra_, while increasing in SC_ipsi_, producing a bilateral imbalance in SC activity. Orange shading indicates period of laser illumination; orange dashed lines indicate laser onset (50-ms before trial start). Black dashed lines and triangles indicate stimulus onset. Orange dashed lines indicate laser onset. SC_contra_ recordings shown in black; SC_ipsi_ in blue. Solid lines and shaded regions indicate mean and s.e.m. across units. n=1 session, SC_contra_: n = 48 single units, SC_ipsi_: n = 33 single units.

Unilateral S1 inactivation can shift choice bias by altering the interhemispheric balance downstream in SC. In support of this idea, simultaneous bilateral SC recordings in a limited dataset revealed that S1_contra_ inactivation not only decreased activity in SC_contra_, but concurrently increased baseline activity in the opposite SC_ipsi_ (Extended Data Fig. 10). This effect is consistent with models of mutual inhibition across SC^44^. Moreover, recent work in primates performing a visual attention task suggested that perceptual choices can be predicted by relative activity between the two SC hemispheres^24^. Cortical inactivation leading to interhemispheric balance is reminiscent of the classic Sprague effect^57^, in which unilateral lesions of the visual cortex led to hemineglect, which could be remediated by an additional lesion in the opposite SC hemisphere. Based on an interhemispheric imbalance model, we speculate that unilateral S1 inactivation may induce an offset between SC hemispheres, altering the amount of sensory evidence required to reach the threshold for a detection response.

Although our results implicate SC as essential for S1 inactivation-induced bias shifts, they do not distinguish whether S1 modulates SC via direct S1-SC projections or indirectly via higher-order sensory or motor cortical areas and subcortical areas such as basal ganglia, thalamus, and the trigeminal brainstem nuclei. Prior work has demonstrated that suppressing activity in S1 axon terminals in either SC, POm, or dorsal striatum can impair detection behavior^11^. In addition, the cortico-basal ganglia circuits have been proposed to regulate decision thresholds by influencing SC output, providing an indirect pathway by which S1 could modulate sensory-guided reports in SC^58^.

Given the convergence of multisensory inputs and capacity for rapid sensorimotor transformations, the SC is ideally suited for rapid detection of salient stimuli in the environment. However, its relatively limited spatiotemporal receptive fields and strong neural adaptation constrain both the spatial and temporal resolution of sensory integration^46,59,60^. Such properties make SC ideally suited for rapid detection, but poorly suited for finer discrimination, sensory evidence accumulation, or complex perceptual judgments. In these contexts, primary and higher-order sensory cortices likely play a more indispensable role, contributing not only to bias adjustments but also to the fidelity of sensory encoding, which is essential for more demanding tasks^61^. For example, in primates, attentional modulation of V4 reflects changes in sensitivity rather than bias^61^, and recent work in primate SC similarly found that attention-related shifts in SC reflect sensitivity and motor bias, but not perceptual bias^62^.

Together, our results suggest that S1 not only enhances sensory responses in SC but also modulates SC to shape decision criteria that guide action selection. Such a mechanism could enable animals to dynamically shift biases in response to context, including reward contingencies, attentional state, and ongoing task demands. For example, adopting a liberal choice bias in response to detecting a looming predator can be life-saving, by increasing detection response probability at the cost of higher false alarms. These results may also help explain how acute cortical perturbations can lead to apparent sensory impairments during detection behaviors, even when the primary sensory cortex is not strictly required for simple detection behaviors^15,10,12,9,11,6^. Rather than merely decreasing sensory information, cortical perturbation may disrupt the balance of downstream decision circuits. Similar principles likely extend beyond the somatosensory system, representing a general mechanism in which cortical areas can flexibly bias subcortical decision circuits.

## METHODS

### Animals.

All animal procedures complied with National Institute of Health Guide for the Care and Use of Laboratory Animals and were approved by the Institutional Animal Care and Use committee at Carnegie Mellon University. For S1 inactivation experiments, *Emx1-IRES-Cre*^35^ knock-in mice (Jackson Laboratories, stock #005628) were crossed to *Rosa-lox-stop-lox (RSL)-eNpHR3.0/eYFP* mice (Ai39, JAX, stock# 006364), which express halorhodopsin (eNpHR3.0) after excision of a stop cassette by Cre recombinase (*Emx1-Halo*, n=19). *Pitx2–Cre*^2^ mice (*Pitx2tm4(cre)Jfm*, provided by J. Martin and N. Kurpios) were crossed to *RSL-H2B–GFP* mice (provided by J. Huang) to express GFP for visualization of Cre-positive neurons (*Pitx2-GFP*, n=1, histology only) or with Ai39 to express halorhodopsin (Pitx2-Halo, n = 6 mice) in a subset of cells in the intermediate layer of SC. All mouse lines were maintained on a C57BL/6J background. Optogenetic experiments used mice that were heterozygous for the transgene. No significant sex differences were found in learning rate, baseline performance, or effect of S1 inactivation between males and females (*Emx1-Halo*, 7M, 5F; *Pitx2-Halo*, 4M 2F). Data from males and females were therefore pooled for analyses.

### Behavioral setup.

Behavioral events were controlled by a microcontroller (Arduino). Water-restricted animals were head-fixed, and a ~4-mm stainless steel tubing (McMasterCarr #8988K79) was temporarily glued (Elmer’s) right C2 whisker, 4–8-mm from the base of the follicle. Remaining whiskers were trimmed to facilitate access to the C2 whisker. A single, gaussian pulse was delivered to an electromagnetic coil (Erse audio) using a 300W audio amplifier at variable amplitudes, resulting in an electromagnetic pulse that deflected the whisker downwards (~25ms from baseline to peak deflection). At the start of each test session, the stimulus amplitude was calibrated with high-speed videography (Basler, 250–500 fps), where the maximum amplitude (amp) 10 was calibrated to ~40°, equivalent to an angular velocity of ~2100 °/s (Extended Data Fig. 1c–d). This maximum whisker velocity is in the range that mice used when voluntarily whisking to detect an object in a previously reported detection task^9^. Two water ports were placed ~0.5cm apart, and licks were detected with an infrared diode-phototransistor (Sparkfun QRD1114). The passive whisker stimulus design enabled precise control over stimulus timing and amplitude, circumventing the confound that S1 inactivation can reduce overall whisking vigor and inadvertently alter the experienced stimulus strength^9^. We implemented a forced-choice (Yes/No) paradigm that requires an overt response on every trial, avoiding the ambiguity inherent to Go/Nogo tasks, where an absence of response for ‘Nogo’ trials could reflect motor or motivational deficits.

### Whisker-mediated tactile detection behavior.

#### Training:

Adult mice (P181 +/− 47 days, mean and SD; 58% male) were implanted with custom-designed headplates with dental cement (Metabond) under isoflurane anesthesia. After at least 3 days of recovery, mice were water restricted to begin training in stages as previously described^2,3^: (1) Habitation: mice were gradually habituated to handling and head-fixation in light-sealed behavior boxes (1–2 days). (2) Dual lick-port training: head-fixed mice learned to lick the two ports equally, by alternating the rewarded port (4d). (3) Simple task: mice then underwent stimulus association training. Trials started with a 50-ms start cue, followed by a stimulus 700-ms from trial start (amplitude 0 or 10 only), where the right port was associated with stimulation of the animal’s right C2 whisker and the left port with no stimulus (2–4d). During training sessions, a response cue tone was delivered 300-ms from stimulus onset, after which lick responses were registered. Mice received a drop of water reward for correct responses, or a 1-s auditory white noise stimulus as punishment for incorrect responses. Learning was defined by a criterion of d’>1.5 for 3 consecutive sessions (~75% correct performance). Mice learned the simple task with a median of 9 sessions (Extended Data Fig. 1a–b). (4) Full task: each session started with 50 warmup trials, during which alternating trials of stimulus amplitude 0 or 10 delivered to habituate animals to the positions of two lick ports. Once animals passed the warmup block, intermediate stimulus amplitudes were added. Each stimulus (amplitudes 0, 3, 4, 6, or 10 AU) was delivered with a probability of 0.4, 0.1, 0.1, 0.2, and 0.2, respectively. Probabilities and amplitudes were determined empirically to produce reliable threshold measurements and stable performance across sessions and animals. To prevent animals from adopting a Go/Nogo strategy, if the animal failed to respond to a single trial type for 4 consecutive trials, a correction block was triggered, in which animals were required to correctly respond for 3 consecutive trials of the non-preferred trial type before continuing the session, and mice continued to train until behavioral performance stabilized before participating in experiments. Warmup and correction block trials were excluded from all analyses.

#### Full task.

For the final Yes/No forced choice paradigm used for experiments, A 50-ms auditory tone signaled the start of each trial, and a stimulus (amplitude 0, 3, 4, 6, or 10 AU) was delivered at 700ms from trial start. After a 300-s delay period, the response window opened for 2-s, and licking the correct water port delivered a drop of water reward (Fig. 1c). The next trial began after a fixed inter-trial-interval (ITI) of 2–3 s. For behavior-only sessions, lick timing was defined as the first registered lick during the response window (data was acquired with an Arduino microcontroller), and should be interpreted as the latency of the first registered lick than a precise measure of reaction time. For behavior during neural recordings, analog lick signals were recorded with the neural data through the OpenEphys data acquisition system, and true lick latencies were quantifiable. With the exception of the start cue tone, all auditory cues (‘go’ cue for response window and punishment sound) were removed to avoid auditory responses in SC recordings. For the Go/Nogo version of the task (Extended Data Fig. 6k–n), animals were trained to lick a single lick port to report stimulus detection (Go), or do nothing if no stimulus was detected (No-go). If the animal licked within 500-ms before the trial start, the trial was aborted.

### Intrinsic signal optical imaging to map C2 barrel in S1.

Functional mapping of the C2 barrel was performed as previously described^9^. Under isoflurane anesthesia, the skull over S1 was thinned (~4mm window centered around stereotactic coordinates AP −1.5, ML 3.5 from Bregma) to clearly visualize the surface vasculature, and sealed with a thin layer of cyanoacrylate. With a camera positioned above the mouse (Teledyne FLIR), a 530 nm LED (Thorlabs) was used to acquire an image of the vasculature, and a 625 nm LED was used to acquire the intrinsic signal. The C2 whisker was stimulated at 10 Hz with a piezo stimulator, and the signal was averaged over 20–30 trials. The signal was then mapped onto the vasculature image. Imaging and analysis were performed using custom written software in MATLAB.

### Optogenetic inactivation.

#### S1 inactivation:

S1 inactivation in *Emx1-Halo* mice was performed as previously described^9^. A 594-nm laser (Coherent) was delivered via a 200-um diameter optic fiber (0.39NA, Thorlabs) positioned over a thinned skull, with the fiber tip centered at the mapped C2 barrel (35mW from fiber tip). Based on our previous characterization of S1 photoinhibition in *Emx1-Halo* mice, neurons within a 1mm radius can be effectively silenced, which fully encompasses the center C2 barrel and extends beyond the adjacent whisker barrels in S1^9^. Laser ON trials were randomly interleaved in 50% of trials. The laser turned on 50-ms before the trial start cue to ensure maximum inactivation by trial start, and remained on for the full duration of the trial. Before the first test session, animals were habituated to the laser without inactivation, with the optic fiber positioned above the mouse’s head (> 10 cm) with blackout tape covering the skull, illuminating the behavioral setup but not affecting neural activity. This also served as negative control to rule out changes in behavior due to visibility or startle effects during laser on trials (**Extended Data Fig. e-i)**. For experiments directly comparing unilateral vs. bilateral S1 (n=6 *Emx1-Halo* mice, 14 sessions) or SC inactivation (n=6 *Pitx2-Halo* mice, 21 sessions), the laser beam was split and directed into 2 optic fiber ports, and calibrated to maintain equal output power (30mW) from each fiber. S1 inactivation was targeted to the center of the mapped C2 barrel coordinates.

#### SC inactivation.

SC illumination was delivered via implanted optic ferrules (AP −3.7, ML 1.7, from Bregma, 1.6–1.9mm depth from pial surface, inserted at a 20° angle from the vertical plane). The laser output from each fiber was balanced and calibrated (30-mW output from each 200um optic fiber tip, 0.39NA) to maintain balanced power levels between hemispheres and across animals and sessions. We used *Pitx2-Cre* knock-in mice^63^ to drive expression of eNpHR3.0 (Ai39 reporter mice) (*Pitx2-Halo*) in excitatory neurons of the SC intermediate layers (SGI). *Pitx2-cre* is expressed in a functionally heterogeneous, glutamatergic population that forms modular premotor units that tile SGI and accounts for around half of local excitatory neurons^38^. Inactivation of excitatory neurons using *Pitx2-Halo* was chosen rather than excitation of inhibitory neurons, which have been shown to generate contralateral choice biases via inter-collicular inhibition^44^, potentially confounding interpretations about local SC computations. *Pitx2-Cre* targeting avoids direct activation of long-range inhibitory neurons, many of which project to and from SC.

### SC lesions.

SC_contra_ was lesioned by laser-mediated ablation, calibrated to target the superficial to intermediate layers of SC while minimizing damage to neighboring structures. Mice were deeply anesthetized under isoflurane, and a large craniotomy was made over SC. To target contralateral SC, an optic fiber (200um, 0.39NA) was sequentially inserted at 3 locations (AP −3.7; ML 1, 1.7, 2.2-mm from Bregma) at different depths (Z −1.14, −1.5, −1.75-mm) from the pial surface. At each location, a 432-nm laser (Laserglow) was applied for 5–10 minutes at each location 100mW. The craniotomy was then sealed with Vetbond (3M) and covered with dental cement (Stoelting).

Due to variability in SC lesion sizes and locations, animals were excluded from analysis when the following histological criteria applied: (i) small lesions that did not encompass at least 300-um of the expected C2-responsive area (Extended Data Fig. 6, Extended Data Fig. 9); (ii) lesions that extended in to lateral SC (known to control lateralized licking movements^21,43,45^); or (iii) lesions that extended beyond the ventral SC border, damaging the midbrain reticular nucleus or periaqueductal gray (PAG). In addition to histological analysis, several behavioral criteria were also used for exclusion: After SC_contra_ lesions, animals were tested for lateralized lick impairments starting 24 hours post-lesion. At the start of each behavioral session, mice were required to alternate licking the right and left water ports for 10 trials to receive water rewards. If animals failed to complete this warm-up period within a few minutes, the session was aborted. Only mice that demonstrated the ability to lick in both directions were tested on the full task for 3 consecutive sessions. Immediately after surgery, some animals exhibited mild ipsiversive circling behavior, a hallmark of unilateral SC lesions, which typically subsided within 1–2 hrs post-surgery. In a few cases, circling behavior was severe and lasted longer, which also corresponded with larger SC lesions extending into the deep SC layers and the medial reticular nucleus. Such animals were excluded from further testing. Histology from all lesioned animals included in the study are shown in Extended Data Fig. 9.

### Histology.

Mice were perfused with phosphate buffer (PB) followed by 4% PFA in PB. Brains were extracted and post-fixed in 4% PFA solution overnight. The tissue was coronally sectioned into 100-um thick slices using a vibratome (Leica) or freezing microtome (Epredia). Sections were mounted and imaged via brightfield or epi-fluorescence microscope (Olympus). An open-source software (github.com/petersaj/AP_histology) was used to align histology sections to the Allen Brain Reference Atlas.

### Electrophysiology.

Topographically aligned C2-responsive areas in S1 and SC were simultaneously recorded during behavior. Under isoflurane anesthesia, a small craniotomy was made over the target area, and silicone gel (Dow) was applied to seal the craniotomy. Between recording sessions, craniotomies were additionally sealed with Kwik-Cast (World Precision Instruments) and re-applied between sessions. To confirm probe locations post-mortem, we applied lipophilic dyes (DiI or DiD, Thermo Fisher Scientific) onto the back of the probes before each recording and histologically confirmed the recording sites. Probe tracts were mapped using Neuropixels trajectory explorer (https://github.com/petersaj/neuropixels_trajectory_explorer) and re-constructed into a 3D model for visualization (Extended Data Fig. 6).

For S1, a linear electrode array (Cambridge Neurotech, 64 channels, 1250-um span, single shank) was inserted into the functionally mapped C2 barrel (see intrinsic optical signal imaging), at a 30° angle to a depth of 1.2-mm from the pial surface to span the full cortical depth. For SC, a two-shank silicon array probe (Cambridge Neurotech 64 channels, 750-um vertical span, 250-um separation between 2 shanks) was inserted at a 20-deg angle. On the first day of recording, probes were targeted to 3.7-mm posterior, 1.7-mm lateral from Bregma; 2.4–2.5mm from the pial surface. The recording target was identified by marching the insertion location of the electrode until robust responses to C2-whisker stimulation were identified in both shanks. Acute recordings were acquired over 3–4 consecutive days. Data was sampled at 30-kHz (OpenEphys acquisition board and software) and band-pass filtered (300–6000 Hz). Spikes were sorted off-line with Kilosort 2.5 or 3.0^64^ and single units were manually curated using open-source Python library Phy (https://github.com/cortex-lab/phy.git). For each unit, spike trains were aligned to trial onset and binned by 1-ms. Only well-isolated single units were used for analysis. Sessions with fewer than 10 units in either area were excluded from analysis. Experiments with no significant stimulus-responsive units in either single area were deemed to be mis-targeted, and were excluded. For all quantification and statistical analyses, firing rates were analyzed without temporal filtering. For visualization of peri-stimulus time histograms (PSTHs), spike data were smoothed with a gaussian kernel (sigma = 3-ms).

### Whisker tracking.

Whisker angles were quantified using high-speed videography (Basler, 250–500 fps) under infrared illumination for each session. DeepLabCut^65^ was used to track whisker movements for each session. The maximum angle deflection during the stimulus window (0–100-ms after stimulus onset) was determined for each trial, and did not differ between laser OFF and ON trials (Extended Data Fig. 1C–D).

### Quantification and statistics

#### Behavioral analysis.

The probability of ‘Yes’ response (P(Yes)) was calculated for each stimulus amplitude (total ‘Yes’ responses/total valid trials at each amplitude). ‘False alarm’ rate is the probability of ‘Yes’ responses to no stimulus (amplitude = 0) trials. ‘Hit rate’ is the probability of ‘Yes’ response for all trials with stimulus > 0. The percent correct was the hit rate + (1- false alarm rate), normalized by equally weighing stimulus and no stimulus trials. For figure display only, P(Yes) plots were fitted with logistic fits of the average probability of ‘Yes’ response at each stimulus amplitude.

To quantify psychometric performance, we used logistic regression analysis:

(1) $$\text{logit}(\text{P}(\text{Yes}))=\beta_{0}+\beta_{1}\times \text{s}$$

where P(Yes) is the probability of a ‘Yes’ response; β_0_ is the intercept (bias); β_1_ is the slope (sensitivity) and **s** is the stimulus amplitude. A decrease in bias a more conservative response strategy (less likely to respond ‘Yes’), while an increase reflects a more liberal decision strategy (more likely to respond ‘Yes’), regardless of the stimulus strength. Detection threshold is equivalent to the stimulus amplitude needed to respond ‘Yes’ 50% of the time (-β_0_/β_1_). Bias measurements were closely correlated with the false alarm rates and consistently reflected behavioral shifts induced by inactivation. In contrast, sensitivity parameter β_1_ was variable and inconsistent, as impaired performance can be accompanied by increases or decreases in slope (simulated in Extended Data Fig. 2 for clarification). This made sensitivity estimates unreliable as a measure of perceptual sensitivity. To address this, we complemented logistic model analysis with model-free measurements of performance, including the raw probability of detection values by stimulus amplitude (p(Yes) curves), which explicitly incorporates the observed false alarm rate (p(Yes | amp 0)). We also derived thresholds from the raw data by interpolation, quantifying the amplitude at which the detection probability crossed 0.5. These interpolated thresholds were highly consistent with model-derived thresholds (-β_0_/β_1_), and provided confirmation that threshold measurements were reliable. Thus, we emphasized bias, false-alarm rates, and interpolated thresholds as stable measurements of decision criteria, while treating the model-derived sensitivity estimates with caution.

For each session, only ‘real’ session trials were included in analysis; warmup trials, correction trials, and non-responded (no licking to either port) trials were omitted. In addition, sessions were excluded if any stimulus amplitude contained no responded trials, or if the session had fewer than 100 responded trials due to early satiation, and guarding against degenerate fits. To ensure good performance, only sessions where OFF-trial performance d-prime (between amplitudes 0 vs. 10) > 1.5 were included (d’ = Z(Hit rate) – Z (False alarm rate), where Z is the inverse of the standard normal cumulative distribution function). Sessions that failed basic fit criteria (p ≥ 0.5 for logistic model fit) were omitted from model-based summaries (threshold, intercept (b1), and slope (b0), but were included in model-independent raw values (p(Yes), % correct, hit rate, false alarm rate, interpolated threshold).

#### Behavioral statistics.

Analyses were performed using custom MATLAB scripts. Normally was tested using the Shapiro-Wilk test. Statistical significance is represented as *P<0.05, **P<0.01, ***P<0.001, or n.s. (not significant, p ≥ 0.05). All paired comparisons between conditions were assessed with two-sided paired t-tests for normal distributions, or two-sided Wilcoxon signed-rank tests for non-normal distributions. Bonferroni correction was applied to adjust for multiple comparisons. For psychometric analyses, we first fit a two-parameter logistic function (Equation (1)) by session and laser condition. We then compared session-level changes (OFF-ON) in bias (β_0_), sensitivity (β_1_), and threshold (-β_0_/β_1_) using linear mixed-effects models with animals as a random intercept.

#### Whisker stimulus-responsive units.

Single units were classified as whisker stimulus-responsive if the average activity during the stimulus window (100-ms from stimulus onset) for amplitude 10 trials were significantly different (p<0.05, paired t-test)from the baseline activity (500-ms window before stimulus onset. While classification of excitatory (regular spiking, RS) and inhibitory (fast-spiking) cells can be performed by standard clustering analysis of spike waveforms in S1, SC neurons are not classifiable using established methods^65^. Thus, for comparing S1 and SC responses, we did not distinguish between RS and FS cells.

#### Neural and behavioral d’.

To compare neural and behavioral discriminability, we first computed an ideal observer measure based on the area under the receiver operating characteristic (AUROC) for classifying amp 0 vs. each stimulus amplitude. Behavioral AUROC was computed at the trial level with MATLAB’s *perfcurve* function. For neural data, evoked spike rates of stimulus-responsive neurons were quantified as mean firing rate in a 100-ms response window, baseline subtracted by the 500-ms pre-stimulus window on the same trial. For each unit and laser condition, AUROC was only computed when at least 5 trials were available for both amp 0 and each stimulus amplitude. To control for class-size imbalances, neural AUROC used balanced class counts between amp0 and each stimulus by random subsampling, averaged over 50 resamples. AUROC values were then converted to a d’ via the signal detection mapping:

(2) $$d_{\text{AUROC}}^{\prime }=\sqrt{2}\phi^{-1}(\text{AUROC})$$

For neural data the sign of d’ was set by the sign of the evoked-rate difference (amp s – amp 0).

#### Mean square error (ΔMSE):

To quantify how well the behavioral change produced by S1 inactivation was captured by the sensory-evoked neural activity in each area, we calculated the AUROC-derived Δd’ = d’_ON_ – d’_OFF_ at each amplitude for behavioral sessions. For neural data, Δd’ was calculated from the mean AUROC-derived d’ curve for each area. To focus on the shape of the inactivation-induced change across stimulus amplitudes, rather than the overall gain or offset, Δd’ curve was min-max normalized within subject and area:

(3) $$\tilde{\Delta d}\prime (k)=\frac{\Delta d\prime (k)-min_{j}\Delta \text{d}\prime (\text{j})}{{\text{max}}_{\text{j}}\Delta \text{d}\prime (\text{j})-min_{j}\Delta \text{d}\prime (\text{j})}$$

We then quantified the shape mismatch between neural and behavioral changes by the mean squared error across amplitudes:

(4) $$\Delta MSE=\frac{1}{K}\sum_{K=1}^{K}{\left(\Delta \tilde{d_{\text{neural}}^{\prime }}(k)-\Delta \tilde{d_{\text{behavior}}^{\prime }}(k)\right)}^{2}$$

where K is the number of non-zero stimulus amplitudes included. A lower ΔMSE indicates a closer match in the pattern of inactivation-induced changes between neural and behavioral d’ curves, independent of absolute scale and offset.

#### Optogenetic Modulation Index (OMI).

For each single unit, the average firing rate within the stimulus window (100-ms window after stimulus onset) at near-threshold amplitude 6 was compared for laser OFF vs. ON conditions. For the OMI of spontaneous activity, a 500-ms window before stimulus onset was used. The optogenetic modulation index was calculated as the difference over the sum of the firing rates for OFF vs. ON trials.

(5) $$\text{OMI}=({\text{FR}}_{\text{ON}}-{\text{FR}}_{\text{OFF}})/({\text{FR}}_{\text{ON}}+{\text{FR}}_{\text{OFF}})$$

where FR is firing rate. Positive values indicate increased activity, while negative values indicate decreased activity. For each unit, significant modulation was determined using an unpaired t-test across trials, and p < 0.05 was used to identify units with significant modulation.

#### Population decoding analysis.

Decoding analyses were performed using custom MATLAB scripts with Statistics and Machine Learning Toolbox and Neural Decoding Toolbox^66^. Linear support vector machines (SVM) were trained to classify stimulus presence vs. absence of stimulus, or behavioral choice (lick right vs. lick left to stimulus). For both stimulus and choice decoding, amplitude 6 was chosen because it represented the near-threshold stimulus where lick right vs. left trials were most balanced. Recordings were performed simultaneously in S1 and SC per session, but eligible units across sessions were pooled within each area. Neural data was binned in 50-ms sliding windows stepped by 25-ms. To minimize activity from licking or motor preparation, analysis was restricted to a 100ms window starting at stimulus onset (700–800-ms from trial start). Units were eligible if they had at least trials per class. For each analysis, the number of units per resample run was down sampled to the minimum number of eligible units across S1 and SC. To facilitate comparison across decoding analyses, the number of units were matched across stimulus and choice decoding analyses (n=282 for OFF only analyses (Fig. 4h–i); n=119 for OFF vs. ON comparisons (Fig. 6e–f)). For cross-condition generalized decoding analysis across OFF and ON trials, classifiers were trained on OFF trials, and tested on held-out OFF or ON trials. Trials were also matched by behavioral response (lick direction) for all decoders. Units were sampled without replacement per resample run, and trials were sampled without replacement per unit and class. Decoding was repeated across 200 independent resamples to average over unit/trial sampling and cross-validation partitions. Data were z-scored and decoding accuracy was evaluated with 5-fold cross-validation. For statistical comparisons across groups, mean accuracy across bins for each resample run was compared using paired t-tests (OFF vs. ON within area; S1 vs. SC within OFF/ON condition). To assess significance above chance, 1000 shuffle-label permutations were performed for to generate a null distribution of decoding accuracies for each time bin. P-values were corrected for multiple comparisons across bins using Benjamini-Hochberg false discovery rate (indicated as # p<0.05; ## p<0.01; ### p<0.001).

## Extended Data

## Supplementary Material

Supplementary Files

This is a list of supplementary files associated with this preprint. Click to download.

- ReportingSummary.pdf

## Data Availability

All data and custom MATLAB code used for analysis will be made available at publication via the Zenodo database (# TBD).
